# Feasibility of laryngeal mask anesthesia combined with nerve block in adult patients undergoing internal fixation of rib fractures: a prospective observational study

**DOI:** 10.1186/s12871-020-01082-y

**Published:** 2020-07-15

**Authors:** Jun Cao, Xiaoyun Gao, Xiaoli Zhang, Jing Li, Junfeng Zhang

**Affiliations:** Department of Anesthesiology, Shanghai Jiao Tong University Affiliated Sixth People’s Hospital, No. 600, Yishan Rd., Shanghai, China

**Keywords:** Laryngeal mask anesthesia, Rib fractures, Thoracic paravertebral block, Erector spinae plane block

## Abstract

**Background:**

The laryngeal mask airway (LMA) is occasionally used in internal fixation of rib fractures. We evaluated the feasibility of general anesthesia with an LMA associated to a thoracic paravertebral block (TPB) and/or an erector spinae plane block (ESPB) for internal fixation of rib fractures.

**Methods:**

Twenty patients undergoing unilateral rib fracture fixation surgery were enrolled. Each patient received general anesthesia with an LMA combined with TPB and/or ESPB, which provided a successful blocking effect. All patients received postoperative continuous analgesia (PCA) with 500 mg of tramadol and 16 mg of lornoxicam, and intravenous injection of 50 mg of flurbiprofen twice a day. Our primary outcomes including the partial pressure of arterial oxygen (PaO_2_) and arterial carbon dioxide (PaCO_2_) were measured preoperatively and on the first day after surgery. Secondary outcomes including the vital signs, ventilation parameters, postoperative numerical rating scale (NRS) pain scores, the incidence of postoperative nausea and vomiting (PONV), perioperative reflux and aspiration, and nerve block-related complications were also evaluated.

**Results:**

Thirteen men and seven women (age 35–70 years) were enrolled. Six (30%) had a flail chest, nine (45%) had hemothorax and/or pneumothorax, and two (10%) had pulmonary contusions. The postoperative PaO_2_ was higher than the preoperative value (91.2 ± 16.0 vs. 83.7 ± 15.9 mmHg, *p* = 0.004). The preoperative and postoperative PaCO_2_ were 42.1 ± 3.7 and 43.2 ± 3.7 mmHg (*p* = 0.165), respectively. Vital signs and spontaneous breathing were stable during the surgery. The end-tidal carbon dioxide concentrations (EtCO_2_) remained within an acceptable range (≤ 63 mmHg in all cases). NRS at T1, T2, and T3 were 3(2,4), 1(1,3), and 0(0,1), respectively. None had PONV, regurgitation, aspiration, and nerve block-related complications.

**Conclusions:**

The technique of laryngeal mask anesthesia combined with a nerve block was feasible for internal fixation of rib fractures.

**Trial registration:**

Current Controlled Trials ChiCTR1900023763. Registrated on June 11, 2019.

## Background

Rib fracture is one of the most common injuries following blunt trauma, occurring in approximately 10% of all trauma patients. Surgical stabilization of rib fractures has been shown to be beneficial in those patients with flail chest and multiple severe displaced fractures [1]. In the past, general anesthesia with endotracheal intubation (ETI) was considered mandatory for rib fracture surgery. However, it might cause ventilator-induced lung injury (VILI) [2] and the patients might also have delayed awakening or even need re-intubation owing to residual general anesthetics [3]. Currently, the enhanced recovery after surgery (ERAS) protocol is well established as a standard of care for several surgeries. LMA anesthesia combined with a nerve block could offer an enhanced recovery owing to the possibility of a fast and coughless extubation and effective postoperative analgesia with less opioid [4]. Therefore, we designed this prospective observational study to evaluate the feasibility of general anesthesia with an LMA associated to regional anesthesia in elderly patients undergoing internal fixation of rib fractures.

## Methods

### Participants

This prospective, observational study was approved by the Ethics Committee of Shanghai Sixth People’s Hospital (2019–53) and was registered at www.chictr.org.cn (ChiCTR1900023763). For this study, 20 patients who were scheduled for surgical reduction and fixation of unilateral isolated rib fractures from June to August 2019 were enrolled. Signed informed consent was obtained from all patients. The inclusion criteria were American Society of Anesthesiologists physical status I and II, age 18–70 years, body mass index (BMI) < 30, preoperative PaO_2_ > 60 mmHg, and preoperative PaCO_2_ < 50 mmHg. The exclusion criteria were difficult airway, esophageal reflux, myasthenia gravis, abnormal coagulation system, gastric ulcer or hemorrhage, allergy to anesthesia-related drugs, asthma or chronic obstructive emphysema, major thoracic vascular injuries, and pregnancy.

### Procedures

Non-invasive blood pressure monitoring, pulse oxygen saturation (SpO_2_) monitoring, and electrocardiography were performed on the patients admitted into the operating room.

**FIG. 1:**
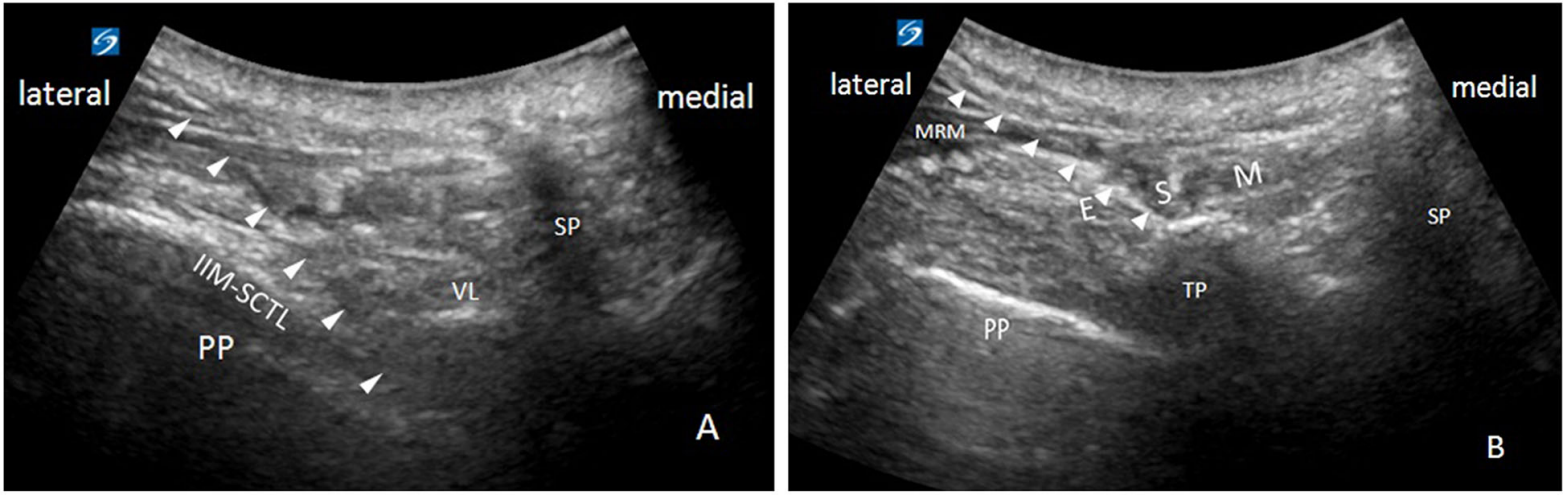
Ultrasound-guided transversal in-plane approach. A, Thoracic paravertebral block. B, Erector spinae plane block. Arrowheads indicate the needle position. PP, Parietal Pleura; VL, Vertebral Lamina; TP, Transverse Process; SP, Spinae Process; IIM-SCTL, Internal Intercostal Membrane, and Superior Costotransverse Ligament; ESM, Erector Spinae Muscle; MRM, Musculus Rhomboideus Major

The ultrasound-guided thoracic paravertebral block (TPB) was performed on the patients who were placed in the lateral decubitus position by using the S-Nerve™ Ultrasound System (Fujifilm SonoSite Inc. Bothell, WA, USA). The transversal inferior articular process (IAP) in-plane approach was applied. A convex array probe (5–2 MHz; C60x; Fujifilm SonoSite Inc. Bothell, WA, USA) was used to visualize the vertebral lamina, internal intercostal membrane, and parietal pleura (Fig. 1a). A 22-gauge, 8-cm puncture needle (KDL medical apparatus and instruments Co. Wenzhou, China) was inserted into the thoracic paravertebral space (TPVS) from the lateral side. Ropivacaine 0.375% (20–30 ml) was injected with no air or blood aspiration.

The injection points of TPVS were selected according to the fractured rib segments requiring surgery (hereinafter referred to as “surgical segments”). If the surgical segments had less than five sequential ribs, 20 ml of ropivacaine was injected into the TPVS of the second fractured rib, referred to as a single-level block; else, 15 ml of ropivacaine was injected into the TPVS of the second and fifth fractured ribs, referred to as a double-level block. We adopted a two-person mode in TPB: one physician operated the ultrasound probe and needle while the other performed the injection and aspiration. Color Doppler ultrasound was initially used to ensure that there were no vessels in the pathway of the needle insertion while approaching the TPVS.

In the case of posterior rib fractures, ESPB was performed to enhance the regional effect of the patient’s back and to supply more effective analgesia of posterior rib fractures as well [5]. Ropivacaine 0.375% (20 ml) was injected between the fifth thoracic vertebral transverse process and erector spinae muscle (ESM) on the operative side using the transversal in-plane approach under ultrasound guidance (Fig. 1b).

The effect of the regional block was evaluated 15 min after nerve blockade, and the dermatomes of sensory loss were measured by acupuncture and rubbing with alcohol gauze. If the patient felt no pain during deep-breathing and vigorous coughing, and the range of reduction area of cold or pinprick sensation covered the incision, we considered the regional effect to be satisfactory. The patient was then given LMA anesthesia and was included in this study. Otherwise, the patient was administered ETI anesthesia and excluded from the observational analysis.

Anesthesia was induced with 0.1 μg/kg sufentanil, 3 mg/kg propofol, and 0.3 mg/kg rocuronium successively. LMA Supreme™ (Teleflex Medical Co. Westmeath, Ireland) was inserted in an accurate position. Mechanical ventilation was initiated with a pressure control ventilation-volume guaranteed mode, at 6 ml/kg and a respiratory rate (RR) of 12 breaths/min. The inspiratory to expiratory ratio was 1:2. A 14# gastric tube was placed for drainage of the fluid and/or gas that might escape into the stomach during positive pressure ventilation.

During the surgery, anesthesia was maintained with sevoflurane at 0.7–1.2 age-adjusted minimum alveolar concentration (MAC) in 50% oxygen in air mixture depending on the hemodynamic responses to surgical intervention. Spontaneous breathing was maintained after recovery. A supplementary dose of 0.03 μg/kg sufentanil was allowed if the HR was 20% faster than the basic value, or RR was more than 20 breaths/min for surgical stimulation. Phenylephrine and atropine were injected if necessary. Sevoflurane inhalation was withdrawn and 50 mg of flurbiprofen was infused intravenously at 15 min before the end of the surgery. The muscle relaxant antagonist and neuromuscular blockade monitoring were not used in this study and all patients were allowed to recover on their own.The case was converted to ETI anesthesia if one of the following occurred during the surgery: 1. The surgical field was difficult to expose because of muscular tension 2. The LMA could not be placed in the correct position after three attempts 3. Hemodynamic instability occurred 4. SpO_2_ was less than 90% or EtCO_2_ was more than 70 mmHg.

PCA (infusion rate 2 ml/h, total volume 100 ml) containing 500 mg of tramadol and 16 mg of lornoxicam was routinely administered to all patients. A dosage of 50 mg of flurbiprofen was infused intravenously twice a day. If the patient’s NRS was > 4, an analgesia rescue of 50 mg of pethidine was administered intramuscularly.

### Data collection

The primary outcomes including PaO_2_ and PaCO_2_ were measured preoperatively and on the first day after surgery.

Secondary outcomes included:

1. vital signs during the anesthesia, tidal volume (Vt), RR, and EtCO_2_ during spontaneous breathing;
2. postoperative time to removal of the LMA and the events of agitation or hoarseness in the post-anesthesia care unit;
3. NRS pain scores assessed at 6 (T1), 12 (T2), and 24 (T3) hours after surgery;
4. incidence of PONV within 48 h after surgery, the perioperative complications such as regurgitation, aspiration, and injuries relating to the nerve block;
5. dosages of sufentanil;
6. the number of cases that were converted to ETI during the operation.

### Statistical analysis

All statistical analyses were performed using SPSS 19.0 software. The sample size was calculated based on the change of PaO_2_. Seventeen patients were required to detect a mean difference of 10 mmHg and standard deviation of 10 mmHg, power of 0.8, and α-value of 0.05. Taking into consideration a potential dropout rate of 15%, we aimed to enroll 20 patients in the study. The values of arterial blood gas analysis, vital signs, ventilation parameters, postoperative extubation time, and NRS pain scores were presented as the mean ± standard deviation, median (interquartile range: min, max), or range (min-max), whichever applicable. Categorical variables such as incidence of PONV, perioperative reflux, aspiration, and nerve block-related complications were expressed as quantitative values or percentages. The results of arterial blood gas analysis measured pre- and postoperatively, were compared using Student’s *t*-test. The significance level was considered as *p* < 0.05.

## Results

**FIG. 2:**
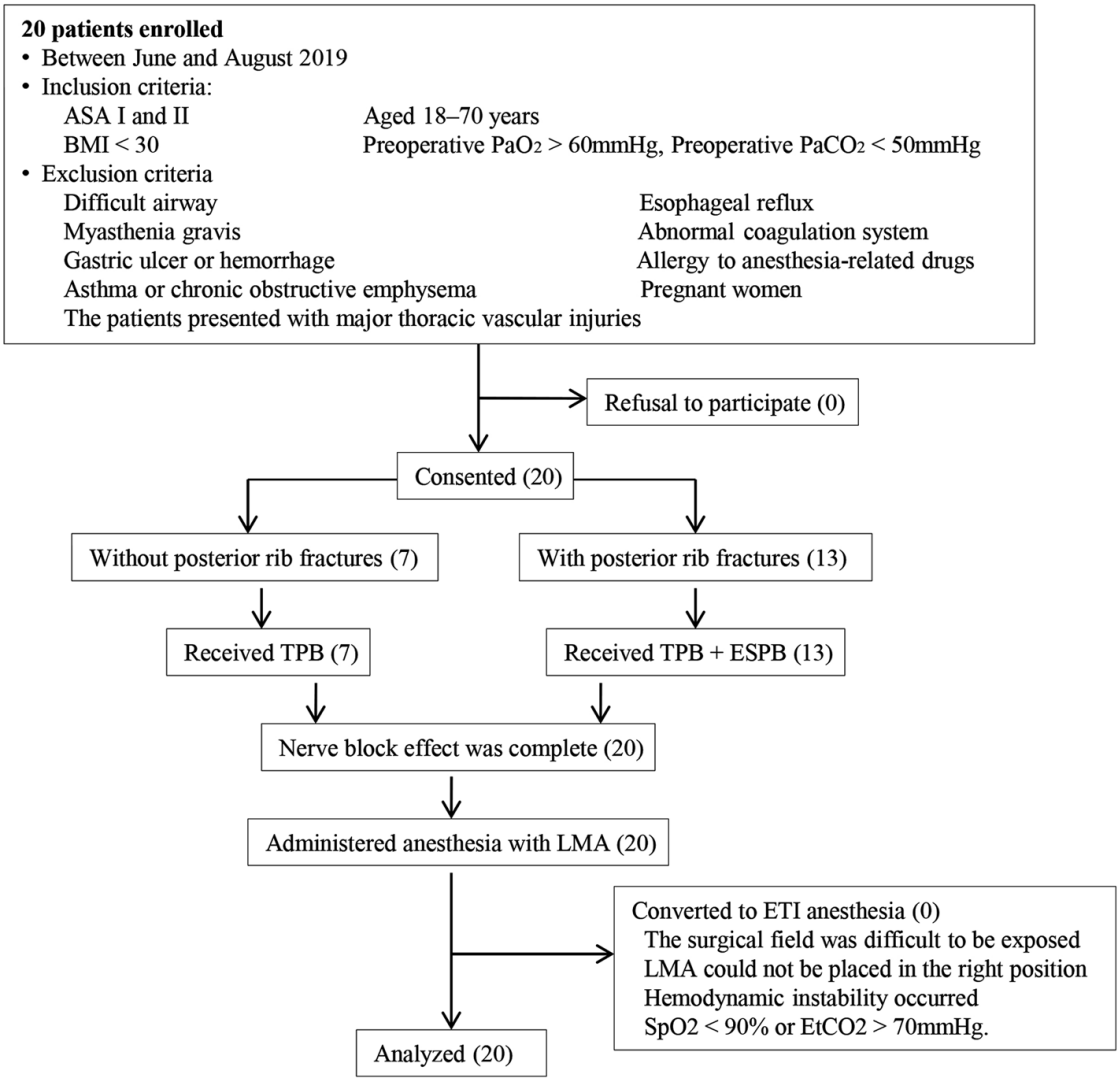
Patients’ flow chart

Twenty patients were enrolled in this study, and their characteristics are listed in Table 1. Of the 20 patients, eight (40%) received single-level TPB, while the remaining 12 (60%) received double-level TPB. Moreover, 13 (65%) patients additionally received ESPB. All patients achieved satisfactory blockade and received LMA anesthesia. No patient required ETI anesthesia owing to the poor position of the LMA or insufficient ventilation. A flow chart of patients recruited for the study is depicted in Fig. 2.

**Table 1:** Demographics and clinical characteristics of the patients

Variable	N	Mean	%
Sex (male/female)	13/7		
Age(y)	35–70	54.15 ± 8.67	
BMI (kg/m ^2^)	19.1–29.7	24.29 ± 2.75	
Flail chest	6		30
Hemothorax and/or pneumothorax	10		50
Atelectasis	5		25
Pulmonary contusion	2		10
Thoracic drainage placed in surgery	9		45
Duration of surgery (min)		70 ± 21

The postoperative PaO_2_ was significantly improved compared to the preoperative value (91.2 ± 16.0 vs. 83.7 ± 15.9 mmHg, *p* = 0.004). Nevertheless, there was no significant difference between preoperative and postoperative PaCO_2_ (42.1 ± 3.7 vs. 43.2 ± 3.7 mmHg, *p* = 0.165).

In most patients, the mean arterial pressure was stable, except for eight (40%) patients, whose MAPs were less than 60 mmHg transiently and were treated by phenylephrine. Patients’ SpO_2_ before anesthesia was 98% (93.25, 98) % and remained above 95% [99% (98, 100) %] during the operation, except for one patient whose SpO_2_ declined from 100 to 87% transiently but recovered to 98% within 5 min. The duration from LMA insertion to spontaneous breathing recovery was 27.3 ± 19.4 min. Vt, RR, and EtCO_2_ during spontaneous breathing were in the range of 205–875 ml, 7–23 breaths/min, 36–63 mmHg, respectively, except for one patient whose EtCO_2_ exceeded 60 mmHg, ranging from 57 mmHg to 63 mmHg. The time to removal of LMA was 6 ± 3 min.

The postoperative NRS scores at T1, T2, and T3 were 3(2,4), 1(1,3), and 0(0,1), respectively. In this study, the highest score was 5 in four patients (20%). Two patients had a score of 5 at 6 h and the other two at 12 h after surgery. All four patients received one intramuscular injection of 50 mg pethidine for rescue analgesia, and pain was relieved. PONV did not occur within 48 h after surgery in all cases.

Sufentanil was administered at a dose of 9.9 ± 3.4 μg. None of the patients developed agitation or sore throat after anesthesia. Perioperative regurgitation, aspiration, and nerve block-related complications were not observed in any of the patients.

## Discussion

In this study, we found that LMA anesthesia combined with nerve blocks such as TPB and ESPB could offer satisfactory analgesia, stable hemodynamic function, good oxygenation, acceptable EtCO_2_, and thereby a smooth recovery.

Although thoracic epidural anesthesia is a gold standard for thoracic analgesia, it can frequently induce hypotension. It is also associated with serious complications such as epidural hematoma and neuropathy [6]. Therefore, various nerve blocks can be used as alternatives to epidural anesthesia, such as the serratus anterior plane block (SAPB), intercostal nerve block (INB), and ESPB. Nevertheless, there are some limitations to the above-mentioned methods. In SAPB, the local anesthetic agent is distributed along the midaxillary line near the surgical incision, which may impede the surgeon from transecting the muscular layers. INB requires multiple injections, subjecting the patient to more pain and increases the risk of inadvertent intercostal vessel or pleural puncture. As ESPB is administered in the intermuscular space, the incidence of a complete block is about 1/3 [7]. As TPB can provide a reliable effect equivalent to that obtained with unilateral epidural anesthesia with lesser hemodynamic depression, we chose TPB for method of regional anesthesia in our study. Meanwhile, all patients maintained steady hemodynamic function with occasional administration of phenylephrine.

The intercostal (IC) and paralaminar (PL) approaches are commonly used in TPB. Yasuko Taketa et al. [8] considered that a single injection of 20 ml 0.375% ropivacaine via the PL approach could acquire 4–5 dermatomes of sensory blockade. They found that the blocked dermatomes of sensory loss were more in the PL group than in the IC group. The average number of blocked dermatomes was three in the IC group and four in the PL group. In addition, the PL approach was regarded as a better choice to block the dorsal ramus of the thoracic nerves [9]. The TPB through a PL approach may obtain a wider block effect compared to the IC approach and should be applied preferentially. Our approach regarding the TPB is the similar to Taketa’s PL approach and is also consistent with the transversal IAP approach described by Krediet et al. [10] Moreover, we chose the TPVS of the second fractured rib for injection when the surgical segments were not more than four sequential ribs because we found that the area of blockade in the caudal direction was more extensive than that in the cephalic direction.

In thoracic surgery, postoperative pulmonary complications (PPCs) are problems that should be addressed. Recruitment maneuver and airway suction might be beneficial to patients during ETI anesthesia. The leak pressure of the LMA Supreme™ was 27.1 ± 5.2 cmH_2_O according to Russo’s study [11]. Thus, the LMA Supreme™ could settle for recruitment maneuver during anesthesia. Early extubation and good analgesia in our study promoted patients to have enough strength to cough and expectorate postoperatively, also beneficial to lung recruitment. In particular, the early recovery of spontaneous breathing reduced PPCs including pneumonia and acute respiratory distress syndrome [12]. Positive pressure ventilation not only changes the pressure gradient of the thoracic cavity and interferes with the distribution of intrapulmonary ventilation, but also leads to an imbalance in the ventilation/perfusion (V/Q) ratio with excessive or inadequate tidal volume. Barotrauma and volume injury caused by mechanical ventilation can also cause VILI. The information above indicates that the spontaneous breathing might be beneficial to lung protection [12, 13]. In our study, the postoperative PaO_2_ was improved compared to the preoperative values.

The patients showed various degrees of carbon dioxide retention during spontaneous breathing. The EtCO_2_ of most patients was below 50 mmHg at the end of surgery. The highest EtCO_2_ value in our study was 63 mmHg and occurred in a male patient whose final EtCO_2_ was 58 mmHg at the end of the surgery. After extubation all patients were fully awake and their PaCO_2_ was normal on the second day after surgery. The concept of permissive hypercapnia has been accepted for a long time. O′ Toole et al. [14] believed that hypercapnia could produce an anti-inflammatory effect by inhibiting nuclear factor-kappa B (NF-κB). Other scholars thought that hypercapnia had a protective effect on VILI [15, 16]. Hypercapnia can also improve pulmonary compliance by a non-surfactant mechanism and enhance pulmonary vascular resistance by strengthening hypoxic pulmonary vasoconstriction to decrease the pulmonary shunt [17].

Most patients with rib fractures experienced dyspnea. The satisfactory effect of TPB could improve patient oxygenation, as respiratory amplitude increases when the patients do not feel pain [18]. The patients’ Vt and RR during spontaneous breathing can meet the needs of intraoperative oxygenation, even in LMA anesthesia with 50% oxygen. Koo et al. concluded that an oxygen concentration of 50% could decrease the risk of atelectasis caused by high oxygen concentration [19]. Our results showed that all patients maintained their SpO_2_ at good level during the operation, including three patients whose preoperative SpO_2_ was lower than 93%. The minimum SpO_2_ was 87% and occurred transiently in one case. Preoperative chest computed tomography showed a large amount of pleural effusion, incomplete atelectasis, and consolidation of the inferior lobe on the injured thorax. The decline in SpO_2_ was attributed to a notable decrease in tidal volume caused by sufentanil. However, it increased to 98% in a few minutes and remained at 100% until the end of surgery.

The serratus anterior and latissimus dorsi muscles were innervated by the long thoracic and thoracodorsal nerves, respectively. TPB cannot paralyze these muscles. We found that one effective dose (ED_95_) of rocuronium could weaken muscle twitching when the surgeons transected the muscles using a high-frequency electrotome. Murphy et al. [3] pointed out that the residual effect of muscle relaxants was one of the causes of postoperative respiratory failure, and critical respiratory events observed in 18.0% of patients undergoing thoracic surgeries. Althausen et al. reported that the incidence of re-intubation after surgical stabilization of the flail chest was 4.55% (1/22) [20]. The half dosage of muscular relaxants for ETI anesthesia in our study facilitated the patients to recover spontaneous breathing during surgery. Therefore, the patients were not monitored for neuromuscular blockade or administer muscle relaxant antagonists. Since SpO_2_ was maintained above 96% during spontaneous respiration at 40% oxygen concentration, each patient was extubated with the confirmation of consciousness, blinking, good swallowing function, and fist clenching in the post-anesthesia care unit. Besides, the patients maintained a good level of cough strength after extubation and did not need re-intubation.

In our study, the analgesic effect of the nerve block was sufficient and maintained for approximately six hours after the operation. This was consistent with the duration of postoperative analgesia of TPB (303.97 ± 76.08 min) reported by Das et al. [21] Due to the PCA and intravenous infusion of flurbiprofen, most patients felt an acceptable level of pain. This indicated that the multi-mode analgesia protocol was effective and necessary for postoperative analgesia. This result also suggests that better postoperative analgesia can be achieved by TPB catheterization in a future study, as reported by Ge et al. [22].

There were several limitations to this study. Owing to the small sample size, the capacity to evaluate potential risks such as regurgitation, aspiration, and nerve block related complications was limited. Patient selection and lack of a control group also contribute to the limitations. Since the initial focus was on whether the anesthetic technique could meet the needs of rib surgery, we excluded patients with complicated extubation conditions such as respiratory failure, obesity, difficult airway, myasthenia gravis, asthma, and chronic obstructive emphysema. Although early extubation occurred and PPCs such as pneumonia and acute respiratory distress syndrome were not observed, these findings are not sufficient to declare superiority when compared with ETI anesthesia. The results in our study might provide a basis for further randomized controlled trials to assess the safety and effectiveness of this anesthesia technique.

## Conclusion

We demonstrated that LMA anesthesia combined with nerve blocks could be feasibly applied in our selective patient population undergoing internal fixation of rib fractures. This practice could provide stable hemodynamic and respiratory function, and the advantage of a smooth recovery. Our protocol could represent a draft of an ERAS anesthetic protocol for this type of surgery.

## Data Availability

The datasets used and analyzed during the current study are available from the corresponding author on reasonable request.

## Abbreviations

LMA: Laryngeal mask airway
TPB: Thoracic paravertebral block
ESPB: Erector spinae plane block
HR: Heart rate
BP: Blood pressure
SpO_2_: Pulse oxygen saturation
PCA: Postoperative continuous analgesia
NRS: Numerical rating scale
EtCO_2_: End-tidal carbon dioxide concentration
PaO_2_: Partial pressure of arterial oxygen
PaCO_2_: Partial pressure of arterial carbon dioxide
ETI: Endotracheal intubation
VILI: Ventilator-induced lung injury
BMI: Body mass index
IAP: Inferior Articular Process
TPVS: Thoracic paravertebral space
PONV: Postoperative nausea and vomiting
Vt: Tidal volume
RR: Respiratory rate
MAC: Minimum alveolar concentration
SAPB: Serratus anterior plane block
INB: Intercostal nerve block
IC: Intercostal
PL: Paralaminar
ED_95_: 95% effective dose
PPCs: Postoperative pulmonary complications
